# Digital Gaming and Exercise Among Youth With Type 1 Diabetes: Cross-Sectional Analysis of Data From the Type 1 Diabetes Exercise Initiative Pediatric Study

**DOI:** 10.2196/57198

**Published:** 2024-06-13

**Authors:** Susana R Patton, Robin L Gal, Simon Bergford, Peter Calhoun, Mark A Clements, Jennifer L Sherr, Michael C Riddell

**Affiliations:** 1 Nemours Children's Health, Jacksonville, FL, United States; 2 Jaeb Center for Health Research, Tampa, FL, United States; 3 Children's Mercy Hospital, Kansas City, MO, United States; 4 Yale School of Medicine, New Haven, CT, United States; 5 Muscle Health Research Centre, York University, Toronto, ON, Canada

**Keywords:** exercise, exercises, exercising, physical activity, physical activities, digital game, digital games, gaming, electronic game, electronic games, computerized game, computerized games, pediatric, pediatrics, child, children, youth, adolescent, adolescents, teen, teens, teenager, teenagers, diabetes, diabetic, DM, diabetes mellitus, type 2 diabetes, type 1 diabetes, TD1, TD2, mobile phone

## Abstract

**Background:**

Regular physical activity and exercise are fundamental components of a healthy lifestyle for youth living with type 1 diabetes (T1D). Yet, few youth living with T1D achieve the daily minimum recommended levels of physical activity. For all youth, regardless of their disease status, minutes of physical activity compete with other daily activities, including digital gaming. There is an emerging area of research exploring whether digital games could be displacing other physical activities and exercise among youth, though, to date, no studies have examined this question in the context of youth living with T1D.

**Objective:**

We examined characteristics of digital gaming versus nondigital gaming (other exercise) sessions and whether youth with T1D who play digital games (gamers) engaged in less other exercise than youth who do not (nongamers), using data from the Type 1 Diabetes Exercise Initiative Pediatric study.

**Methods:**

During a 10-day observation period, youth self-reported exercise sessions, digital gaming sessions, and insulin use. We also collected data from activity wearables, continuous glucose monitors, and insulin pumps (if available).

**Results:**

The sample included 251 youths with T1D (age: mean 14, SD 2 y; self-reported glycated hemoglobin A_1c_ level: mean 7.1%, SD 1.3%), of whom 105 (41.8%) were female. Youth logged 123 digital gaming sessions and 3658 other exercise (nondigital gaming) sessions during the 10-day observation period. Digital gaming sessions lasted longer, and youth had less changes in glucose and lower mean heart rates during these sessions than during other exercise sessions. Youth described a greater percentage of digital gaming sessions as low intensity (82/123, 66.7%) when compared to other exercise sessions (1104/3658, 30.2%). We had 31 youths with T1D who reported at least 1 digital gaming session (gamers) and 220 youths who reported no digital gaming (nongamers). Notably, gamers engaged in a mean of 86 (SD 43) minutes of other exercise per day, which was similar to the minutes of other exercise per day reported by nongamers (mean 80, SD 47 min).

**Conclusions:**

Digital gaming sessions were longer in duration, and youth had less changes in glucose and lower mean heart rates during these sessions when compared to other exercise sessions. Nevertheless, gamers reported similar levels of other exercise per day as nongamers, suggesting that digital gaming may not fully displace other exercise among youth with T1D.

## Introduction

Type 1 diabetes (T1D) is a common chronic medical condition among children [1]. It is characterized by a loss of endogenous insulin production in the pancreas and an inability to self-regulate blood glucose levels. The daily treatment for T1D includes vigilant glucose monitoring, carbohydrate counting, and intensive insulin delivery, with the goal of trying to achieve near-normal glucose levels [2]. Additionally, it is important for youth living with T1D to participate in regular physical activity and exercise as part of achieving a healthy lifestyle [3]. Current clinical recommendations guide youth living with T1D to achieve at least 60 minutes of moderate- to vigorous-intensity physical activity daily [4], which are the same recommended duration, intensity level, and frequency of physical activity as those recommended for youth without T1D [5,6]. However, results of a recent meta-analysis showed that many youth living with T1D do not meet the minimum recommended levels of physical activity and may even be less physically active than peers without T1D [7].

For all youth, regardless of disease status, minutes of physical activity compete with other daily activities, including school, extracurricular activities, sleep, meals, and socializing [8]. Playing digital games is another popular leisure activity for many youth [9]. Digital games represent any game that is played by using an electronic device. In 2021, it was estimated that the typical US teenager spent almost 2 hours per day playing digital games [10]. Although there is an emerging body of research examining whether playing “serious digital games” could be an effective method of preventing disease in youth [11-14], studies have also explored whether digital games could be displacing other physical activities and exercise among youth [15], thereby potentially contributing to poorer health outcomes [10]. To date, the association between digital games and daily physical activity minutes among youth with T1D has received no attention.

The Type 1 Diabetes Exercise Initiative Pediatric (T1DEXIP) study is a real-world observational study of physical activity and glycemic levels in youth living with T1D from across the United States [16]. The study collected youth-specific (eg, age, insulin regimen, and hypoglycemia fear) and event-specific (eg, type, duration, and intensity of activity) data that were hypothesized to influence the acute glycemic response to physical activity in youth living with T1D. In this study, we used data from the T1DEXIP study to (1) examine characteristics of youth-identified digital gaming sessions versus nondigital gaming exercise sessions (other exercise) and (2) test whether youth with T1D who played digital games regularly (at least 30 min/day; ie, gamers) engaged in less other exercise than youth who did not play digital games (ie, nongamers). Moreover, related to our second aim, we examined whether gamers identified more barriers to physical activity and fear of hypoglycemia than nongamers. We sought to be the first to examine these important factors for youth living with T1D to inform the pediatric diabetes literature and guide in-clinic exercise consultations among youth living with T1D.

## Methods

### Ethical Considerations

The Jaeb Center for Health Research Institutional Review Board approved all study-related materials prior to participant recruitment. We obtained parent informed consent and participant assent electronically before screening and data collection. Data collection lasted from October 6, 2021, to December 17, 2022. We identified youth’s data by using a study-specific identification number. We compensated youth up to US $100 for their participation.

### Procedures

We recruited participants from pediatric diabetes centers across the United States and through diabetes community conferences, workshops, and web-based platforms. Eligible youth were aged 12 to 17 years, had a T1D diagnosis, were on an intensive insulin regimen (open-loop insulin pump, hybrid closed-loop pump, or multiple daily injections), and spoke English. Youth and parents completed web-based surveys. For 10 days, youth logged any exercise session that lasted >10 minutes, any digital gaming activities, 3 days of food intake, and insulin dosing (multiple daily injections only) in a study-specific version of the Bant Diabetes smartphone app (University Health Network and the Hospital for Sick Children). To objectively measure blood glucose levels, insulin use, and physical activity, we collected continuous glucose monitor (CGM), insulin pump (if available), and wrist-worn activity tracker (Garmin vívosmart 4; Garmin International Inc) data from youth.

### Measures

Youth completed a study-specific demographic and medical history survey to report their age, race, ethnicity, sex, contact information, and diabetes history (including duration of T1D, insulin delivery method, previous occurrences of severe hypoglycemia or diabetic ketoacidosis, and most recent glycated hemoglobin A_1c_ [HbA_1c_] level). To measure youth’s perceptions of barriers to physical activity, they completed the Barriers to Physical Activity in Type 1 Diabetes (BAPAD1) scale [17]. The BAPAD1 scale is a 12-item survey that was validated for use in youth living with T1D. BAPAD1 scale items include general barriers to physical activity (ie, bad weather and school schedule), as well as potential diabetes-specific barriers (ie, risk of hypoglycemia or hyperglycemia). Youth responded to items by using a 7-point Likert scale (1: extremely unlikely; 7: extremely likely), with higher scores indicating the perception of more barriers to physical activity. To measure youth’s fear of hypoglycemia, they completed the Hypoglycemia Fear Survey-Child version (HFS-C) [18,19]. This 25-item survey, which was validated for youth with T1D, measures hypoglycemia fear based on the youth’s level of worry about hypoglycemia and use of hypoglycemia avoidance behaviors. The HFS-C uses a 5-point Likert scale (0: never; 4: almost always). Higher scores on the HFS-C reflect greater levels of hypoglycemia fear. To measure parents’ fear of their child experiencing hypoglycemia, parents completed the Hypoglycemia Fear Survey-Parent version (HFS-P) [19,20]. This is also a 25-item survey, which was designed to measure fear based on the parent’s level of worry about hypoglycemia and use of avoidance behaviors. The HFS-P uses the same response scale as the HFS-C. Higher scores on the HFS-P also reflect greater levels of parent-reported hypoglycemia fear.

### Statistical Analysis

Analyses compared exercise characteristics during digital gaming sessions and other exercise sessions, changes in glucose during digital gaming sessions and other exercise sessions, and mean heart rates during digital gaming sessions and other exercise sessions. An additional analysis compared digital gaming sessions to walks and low-intensity exercise sessions. The calculation of changes in glucose during a digital gaming session and an other exercise session required a CGM reading at the start and end of the session, and mean heart rate calculation required at least 15 minutes of heart rate readings. Analyses also evaluated participant characteristics, exercise characteristics, average changes in glucose during other exercise sessions, and mean heart rates during other exercise sessions for gamers versus nongamers. We completed the analyses by using SAS software, version 9.4 (SAS Institute Inc).

## Results

### Sample Characteristics

Our sample included 251 youths with T1D. Overall, youth were aged a mean of 14 (SD 2) years; they reported a mean HbA_1c_ level of 7.1% (SD 1.3%), a mean diabetes duration of 5.3 (SD 2.9) years, and a mean physical activity level of 2.7 (SD 0.6) on the Physical Activity Questionnaire [21]; and 105 (41.8%) youths were female. Youth logged 123 digital gaming sessions and 3658 other exercise (nondigital gaming) sessions over the 10-day period (Table 1). Types of other exercise sessions that youth reported included walking, basketball, gym class, playing with friends, cycling, running and jogging, swimming, baseball and softball, soccer, and volleyball.

**Table 1.** Comparison of digital gaming and other exercise sessions.

		Digital gaming exercise sessions	Other exercise sessions
Number of exercise sessions		123	3658
Exercise duration (min), median (IQR)		60 (35-115)	40 (20-75)
Glucose at start of exercise (mg/dL), mean (SD)		154 (61)	163 (66)
Change in glucose (mg/dL), mean (SD)		–3 (54)	–15 (58)
Heart rate during exercise (beats/min), mean (SD)		99 (18)	109 (16)
**Exercise time of day, n (%)**			
	Night (12 AM to <6 AM)	0 (0)	21 (0.6)
	Morning (6 AM to <12 PM)	15 (12.2)	914 (25)
	Afternoon (12 PM to <6 PM)	53 (43.1)	1984 (54.2)
	Evening (6 PM to <12 AM)	55 (44.7)	739 (20.2)
**Exercise intensity, n (%)**			
	Low	82 (66.7)	1104 (30.2)
	Medium	39 (31.7)	2150 (58.8)
	High	2 (1.6)	404 (11)

### Characteristics of Gaming Versus Other Exercise Sessions

**Figure 1.**
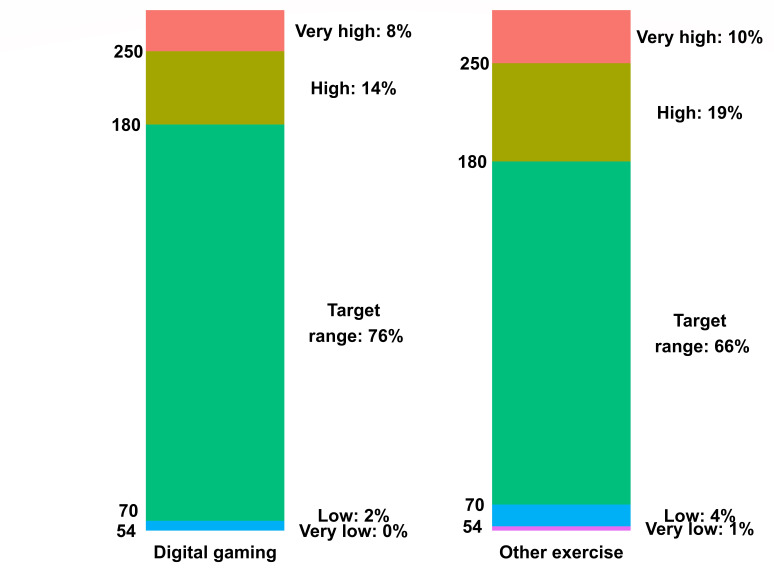
Glucose metrics for digital gaming and other exercise sessions. The percentages on the right of each bar indicate the average percentage of time spent in each glucose category. The numbers to the left of each bar specify glucose levels in mg/dL.

The median duration of digital gaming and other exercise sessions was 60 (IQR 35-115) minutes and 40 (IQR 20-75) minutes, respectively. Prior to digital gaming, youth’s mean glucose level was 154 (SD 61) mg/dL, and prior to other exercise sessions, youth’s mean glucose level was 163 (SD 66) mg/dL. Youth’s mean glucose change was –3 (SD 54) mg/dL during digital gaming and –15 (SD 58) mg/dL during other exercise sessions. When compared to other exercise sessions, during digital gaming, the percentage of glucose time in range (70-180 mg/dL) was higher, and youth experienced slightly less hyperglycemia and hypoglycemia (Figure 1). Youth’s mean heart rate was 99 (SD 18) beats per minute (BPM) during digital gaming and 109 (SD 16) BPM during other exercise sessions. Youth’s mean heart rate during sedentary periods that occurred at the same time as digital gaming but on different days was 90 (SD 12) BPM. Youth described a greater percentage of digital gaming sessions as low intensity (82/123, 66.7%) when compared to other exercise sessions (1104/3658, 30.2%); however, youth described digital gaming as similar in intensity to walking (Table 2). Additionally, when compared to walking and low-intensity exercise, youth had less changes in glucose and lower mean heart rates during digital gaming, digital gaming sessions had longer durations, and digital gaming was more likely to take place during the evening.

**Table 2.** Digital gaming versus walking versus low-intensity exercise.

		Digital gaming exercise sessions	Walking	Low-intensity exercise
Number of exercise sessions		123	975	503
Exercise duration (min), median (IQR)		60 (35-115)	30 (15-45)	40 (25-60)
Glucose at start of exercise (mg/dL), mean (SD)		154 (61)	168 (68)	162 (66)
Change in glucose (mg/dL), mean (SD)		–3 (54)	–16 (53)	–16 (54)
Heart rate during exercise (beats/min), mean (SD)		99 (18)	107 (15)	106 (15)
**Exercise time of day, n (%)**				
	Night (12 AM to <6 AM)	0 (0)	0 (0)	2 (0.4)
	Morning (6 AM to <12 PM)	15 (12.2)	266 (27.3)	121 (24.1)
	Afternoon (12 PM to <6 PM)	53 (43.1)	527 (54.1)	258 (51.3)
	Evening (6 PM to <12 AM)	55 (44.7)	182 (18.7)	122 (24.3)
**Exercise intensity, n (%)**				
	Low	82 (66.7)	601 (61.6)	503 (100)
	Medium	39 (31.7)	367 (37.6)	0 (0)
	High	2 (1.6)	7 (0.7)	0 (0)

### Comparing Gamers to Nongamers

Our sample included 31 gamers and 220 nongamers who were similar in age, insulin regimen, HFS-C total score, HFS-P total score, and BAPAD1 scale score (Table 3). In contrast, we observed a greater percentage of male youths (26/31, 83.9%) in our gamer sample versus our nongamer sample (120/220, 54.5%). Gamers recorded 86 (SD 43) minutes of other exercise per day, whereas 80 (SD 47) minutes were reported by nongamers (Table 4). A follow-up correlation confirmed that there was no association between minutes of digital gaming and minutes of other exercise per day among gamers. The average glucose level at the start of other exercise sessions was 167 (SD 73) mg/dL for gamers versus 163 (SD 66) mg/dL for nongamers, and the mean glucose change during other exercise sessions was –17 (SD 60) mg/dL versus –15 (SD 58) mg/dL, respectively. Exercise characteristics and mean heart rates during other exercise sessions were similar for gamers and nongamers.

**Table 3.** Participant characteristics for gamers and nongamers.

		Gamers (n=31)	Nongamers (n=220)
Age (y), mean (SD)		14 (2)	14 (2)
**Sex, n (%)**			
	Female	5 (16.1)	100 (45.5)
	Male	26 (83.9)	120 (54.5)
BMI percentiles (%), mean (SD)		69 (27)	61 (27)
**Insulin modality, n (%)**			
	Multiple daily injections	4 (12.9)	34 (15.5)
	Open-loop pump	12 (38.7)	64 (29.1)
	Hybrid closed-loop pump	15 (48.4)	122 (55.5)
HFS-P^a^ total score, mean (SD)		46 (13)	41 (13)
HFS-C^b^ total score, mean (SD)		38 (18)	38 (13)
BAPAD1^c^ scale score, mean (SD)		1.9 (0.9)	2.1 (1.0)
Minutes of digital gaming exercise per day, median (IQR)		19 (6-35)	0 (0-0)
Percentage of days with digital gaming exercise, median (IQR)		20 (10-40)	0 (0-0)
Minutes of other exercise per day, mean (SD)		86 (43)	80 (47)
Percentage of days with other exercise, median (IQR)		80 (70-100)	80 (70-90)

a HFS-P: Hypoglycemia Fear Survey-Parent version.

b HFS-C: Hypoglycemia Fear Survey-Child version.

c BAPAD1: Barriers to Physical Activity in Type 1 Diabetes.

**Table 4.** Summary of other exercise sessions for nongamers and gamers (percentages were calculated by using the number of other exercise sessions as the denominator).

		Gamers (n=31)	Nongamers (n=220)
Number of other exercise sessions		468	3190
Exercise duration (min), median (IQR)		40 (20-70)	40 (20-80)
Minutes of exercise per day, mean (SD)		86 (43)	80 (47)
Glucose at start of exercise (mg/dL), mean (SD)		167 (73)	163 (66)
Change in glucose (mg/dL), mean (SD)		–17 (60)	–15 (58)
Heart rate during exercise (beats/min), mean (SD)		111 (16)	108 (16)
**Exercise time of day, n (%)**			
	Night (12 AM to <6 AM)	4 (0.9)	17 (0.5)
	Morning (6 AM to <12 PM)	101 (21.6)	813 (25.5)
	Afternoon (12 PM to <6 PM)	275 (58.8)	1709 (53.6)
	Evening (6 PM to <12 AM)	88 (18.8)	651 (20.4)
**Exercise intensity, n (%)**			
	Low	131 (28)	973 (30.5)
	Medium	308 (65.8)	1842 (57.7)
	High	29 (6.2)	375 (11.8)

## Discussion

In this study’s cohort of physically active youth with T1D, digital gaming sessions lasted longer and were more likely to be described as low intensity, relative to activities such as walking, playing sports, and other physically active social activities that youth engaged in on a regular basis. We also found differences in mean heart rates, changes in glucose, and glucose metrics during digital gaming versus during other exercise sessions. The slightly higher percentage of glucose time in range (70-180 mg/dL) during digital gaming versus other exercise sessions could have been due to the lower starting glucose levels and the little change in glucose during digital gaming. Although the mean heart rate during digital gaming was lower than that during other exercise sessions, it was higher than that during sedentary periods that occurred at the same time as digital gaming but on different days, which is consistent with existing data [22]. Also consistent with existing evidence are youth’s perceptions of digital gaming intensity. In an accelerometry study, researchers characterized digital gaming as similar in intensity to walking or light jogging [23]. However, our study of free-living exercise among youth with T1D offers new results; we reported differences in changes in glucose during exercise and differences in mean heart rates when comparing digital gaming sessions, walking, and low-intensity exercise. We believe that these novel results can help to inform in-clinic exercise consultations among youth with T1D, as youth, parents, and health care professionals may otherwise be unaware of how these activities, particularly digital gaming, may impact glucose levels and heart rates in youth [24,25].

Youth with T1D who reported digital gaming also participated in other exercise. Moreover, comparing daily minutes of other exercise between our gamers and nongamers revealed no differences. This result is consistent with a Dutch study of youth without T1D, which found that the time youth spent playing digital games did not replace the time spent in other physical activities [15]. When comparing gamers to nongamers, there were no differences in hypoglycemia fear or perceived barriers to physical activity. In general, adolescents with T1D report few barriers to physical activity [26], and only hypoglycemia avoidance behaviors [27] appear to be associated with their physical activity.

As an important limitation, this analysis of T1DEXIP study data was likely underpowered to detect differences. Gamers accounted for only 12.4% (31/251) of our sample, which was lower than expected, and this could be an indication of underreporting. Relatedly, because the T1DEXIP study recruited a physically active sample of youth with T1D, it is possible that daily minutes of other exercise may not generalize to typical youth living with T1D. Therefore, we need future research to confirm our study results in a large sample of youth with T1D who report more diverse levels of daily physical activity. It would also be valuable to recruit a large sample of youth with T1D based on their digital gaming habits to determine if minutes of other exercise sessions differ for youth with T1D who self-identify as novice gamers and those who self-identify as avid gamers. We acknowledge the limitation of a possible Hawthorne effect. Youth who participated in the T1DEXIP study were aware that we were interested in examining the associations between their daily physical activity and glycemic levels; therefore, it is possible that they may have altered their gaming and exercise frequencies during the 10-day observation window. Strengths of this study include its real-world observational design and the large number of exercise sessions captured within a large sample of youth with T1D. We also believe that our methods are strong because we used validated surveys and, when available, objective and noninvasive data sources (eg, CGMs, Garmin wearables, and insulin pumps) [28].

In conclusion, for a physically active sample of youth with T1D, our results identified some differences in the duration and intensity of digital gaming versus other exercise sessions, as well as some differences in the mean heart rates and glucose changes during digital gaming versus other physical activity sessions, and suggested that playing digital games may not displace daily minutes spent engaged in other exercise for youth with T1D who are regular gamers. In light of the similarity in daily minutes spent engaged in other exercise between youth with T1D who identify as gamers and those who identify as nongamers, it is possible that interventions for promoting physical activity among youth with T1D should focus on reducing the impact of other barriers to physical activity (eg, hypoglycemia risk and sedentary screen time) rather than target digital gaming.

## Abbreviations

BAPAD1: Barriers to Physical Activity in Type 1 Diabetes
BPM: beats per minute
CGM: continuous glucose monitor
HbA_1c_: glycated hemoglobin A_1c_
HFS-C: Hypoglycemia Fear Survey-Child version
HFS-P: Hypoglycemia Fear Survey-Parent version
T1D: type 1 diabetes
T1DEXIP: Type 1 Diabetes Exercise Initiative Pediatric
